# ‘Cryo-EM’: electron cryomicroscopy, cryo electron microscopy or something else?

**DOI:** 10.1107/S2052252523006759

**Published:** 2023-09-01

**Authors:** Richard Henderson, Samar Hasnain

**Affiliations:** a MRC Laboratory of Molecular Biology, Cambridge CB2 0QH, United Kingdom; b Department of Biochemisty and Systems Biology, University of Liverpool, Liverpool L69 7ZB, United Kingdom

**Keywords:** cryoEM, electron cryomicroscopy, cryo electron microscopy, nomenclature, standardization

## Abstract

Structural biology continues to benefit from an expanding toolkit, which is helping to gain unprecedented insight into the assembly and organization of multi-protein machineries, enzyme mechanisms and ligand/inhibitor binding. During the last ten years, cryoEM has become widely available and has provided a major boost to structure determination of membrane proteins and large multi-protein complexes. Many of the structures have now been made available at resolutions around 2 Å, where fundamental questions regarding enzyme mechanisms can be addressed. Over the years, the abbreviation cryoEM has been understood to stand for different things. We wish the wider community to engage and clarify the definition of cryoEM so that the expanding literature involving cryoEM is unified.

## Abstract 2

The ongoing debate surrounding the acronym ‘cryo-EM’ is considered.

During the review process of a paper that appeared on 9 June 2023 in *Nature Communications* (https://doi.org/10.1038/s41467-023-39140-x), a reviewer raised an interesting comment regarding what the acronym ‘cryo-EM’ stands for. The comment was ‘the correct term is electron cryomicroscopy, not cryo electron microscopy, because the electrons are not cryo electrons, but the microscopy is done at cryo temperatures’. Ironically, three of the references in the manuscript including those published in the *Nature* family of journals had used ‘cryo-electron microscopy’ in their titles, but the reviewer may not have known that this terminology had already been adopted as standard practice by several journals.

Given the growth of cryoEM and continued improvement in resolution (see Fig. 1 ▸), it is clear that cryoEM is here to stay as a major method for structure determination of macromolecules and their complexes. Nearly 14 000 structures have already been deposited, many of them are for membrane proteins and larger assemblies. Some 200 structures have now been made available at resolutions better than 2.2 Å, where atomic details relevant to the transfer of protons or electrons and their regulation become apparent and so fundamental questions regarding enzyme mechanisms can be addressed. It is thus important to clarify the definition of cryoEM so that the expanding literature involving cryoEM is not littered with confusion. It also is important for the new generation of scientists that are entering the exciting world of cryoEM structural biology.

First, it is important to recall how the meaning of cryoEM evolved. The term cryoEM arose from abbreviating cryo-electron microscopy when it became possible to maintain the sample grids in the microscope at cryogenic temperatures. The use of cryoEM spread widely before it was pointed out that electrons were not cold and were not at cryogenic temperature. The community including RH has continued to use cryoEM as an abbreviation for both cryo-electron microscopy and electron cryo-microscopy. Examples of this can be seen in a perspectives article in *PNAS* in 2013 (Henderson, 2013 ▸) and coverage of the Nobel prize in 2017 by *Nature* with the headline ‘Chemistry prize hails work on cryo-electron microscopy’ (Cressey & Callaway, 2017 ▸), both journals having imposed this during editing.

It is now clear that electron cryo-microscopy should not be abbreviated to ‘cryo-EM’; an exact abbreviation would have been ‘E-cryoM’. In our view neither terms ‘cryo electron microscopy’ or ‘electron cryomicroscopy’ are accurate since they imply that either the electrons or the microscope are cold.

In the paper cited in the first sentence (Flynn *et al.*, 2023 ▸), it was suggested that cryoEM should be standardized to stand for ‘single-particle **e**lectron **m**icroscopy with a **cryo**genic sample stage (cryoEM)’, but an alternative and more succinct wording is ‘**cryo**genic-sample **E**lectron **M**icroscopy (cryoEM)’.

Through this letter, we ask the community to consider whether there is a need to find a new consensus for defining succinctly what cryoEM stands for. If so, and a reasonable conclusion can be reached, this could then be put to the IUCr nomenclature commission to consider its formal adoption.

## Figures and Tables

**Figure 1:**
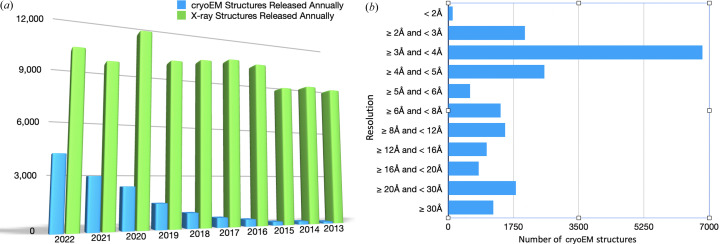
(*a*) Growth of cryoEM structures during the last 10 years. In 2022 the number of deposited cryoEM structures approached over 4000 compared with around 9800 X-ray structures. cryoEM structures were mostly of membrane proteins and large complexes. (*b*) Resolution of cryoEM structures that are now available, nearly 200 structures are now known to ∼2.2 Å resolution.
